# Cost-minimization modeling of venous thromboembolism diagnostics: performing limited compression ultrasound in primary health care reduces costs compared to referring patients to a hospital

**DOI:** 10.1186/s13089-021-00227-5

**Published:** 2021-05-27

**Authors:** Ossi Hannula, Anssi Mustonen, Suvi Rautiainen, Ritva Vanninen, Harri Hyppölä

**Affiliations:** 1 University of Eastern Finland, Kuopio, Finland; 2 Turku University Hospital, Turku, Finland; 3 Pihlajalinna Medical Centre Eastern Finland, Kuopio, Finland; 4 Department of Clinical Radiology, Kuopio University Hospital, Kuopio, Finland; 5 Institute of Clinical Medicine, School of Medicine, University of Eastern Finland, Kuopio, Finland; 6 Emergency Department, South Savo Central Hospital, Mikkeli, Finland

**Keywords:** DVT, POCUS, LCUS, Cost minimization, Primary health care

## Abstract

**Background:**

The aim of this retrospective study was to determine whether diagnosing a deep venous thrombosis (DVT) in primary health care using limited compression ultrasound (LCUS) can save resources compared to referring these patients to hospital. According to the current literature, LCUS is as safe as a standard protocol based on a whole-leg ultrasound (US).

**Methods:**

We created a standardized patient for this cost-analysis model based on 76 patients that were referred to hospital for a suspected DVT. Travel distance to the health care centre and hospital was calculated based on the home address. Hospital costs were acquired from the hospital price list and Finnish legislation. Time spent in the hospital was retrieved from hospital statistics. Time spent in the health care centre and travelling were estimated and monetized based on average salary. The cost of participating physicians attending a US training course was estimated based on the national average salary of a general practitioner as well as the course participation fee. A cost-minimization modeling was performed for this standardized patient comparing the total costs, including private and public costs, of standard and LCUS strategies.

**Results:**

The total costs per patient of standard and LCUS pathways were 1151.52€ and 301.94€ [difference 849.59€ (95% CI 800.21€–898.97€, *p* < 0.001)], respectively. The real-life costs of these strategies, considering that some patients are probably referred to hospital every year and including training costs, are 1151.53€ and 508.69€ [difference 642.84€ (95% CI 541.85€–743.82€)], respectively.

**Conclusion:**

Using LCUS in diagnosing DVT in primary health care instead of referring these patients to the hospital is shown to save a significant amount of public and private resources.

**Supplementary Information:**

The online version contains supplementary material available at 10.1186/s13089-021-00227-5.

## Background

Lower extremity deep venous thrombosis (DVT) is a relatively common disease encountered both in hospital patients and outpatients. It has an annual incidence of 1.2–1.6 per 1000 inhabitants [1, 2]. The most serious complication of DVT is pulmonary embolism (PE) which is often considered as one pole of a continuum of the same disease. In addition to the risk of death associated with PE, DVT frequently causes a post-thrombotic syndrome leading to an impaired quality of life [3, 4]. Hence it is important to correctly diagnose DVT.

Acute DVT is challenging to diagnose due to its non-specific symptoms including sub-febrile fever, pain, swelling and impaired function, which are often associated with other causes. Hence, it is impossible to make the diagnosis on only a clinical basis. The standard method in DVT diagnostics is compression ultrasound (US) with or without Doppler. Venography, computed tomography (CT) and magnetic resonance imaging (MRI) are used only in rare special cases. The sensitivity and specificity of US in diagnosing a proximal DVT are good, 93.8% and 97.8%, respectively, but the sensitivity decreases to 56.8% below the popliteal vein. The use of Doppler seems to lower the specificity to 94% but increases the sensitivity to 96.5% in proximal and 71.2% in distal veins [5]. A single negative US is considered safe in excluding a DVT since only 0.5% of these patients experience a thromboembolic complication during the 3 month follow-up [6]. If the initial US is negative and the symptoms become worse, or the US is technically inadequate, a repetitive US after 5–7 days is recommended [7].

The US examination is traditionally performed by a radiologist and often that requires that the patient needs to be referred to a hospital emergency department (ED). As the symptoms of a DVT are non-specific, the need for US and a hospital visit are frequently expensive, consuming both public and private resources including a loss of working time. By incorporating serum D-dimer testing into the clinical risk assessment based on symptoms and the patient’s history, namely (modified) Wells’ criteria, then the need for a US examination can be reduced by 23% [8]. This can be safely reduced by a further 15% using an age-adjusted D-dimer cut-off point [9].

An alternative method to whole-leg compression US is a limited compression ultrasound (LCUS) examination where only common femoral, proximal superficial femoral and popliteal veins are evaluated. In this approach, LCUS is considered positive if either the thrombus is clearly visualized or the vein is not fully compressible [10–12]. LCUS should be combined with a clinical risk assessment [(modified) Wells’ criteria] and D-dimer [13]. Although the accuracy of LCUS is lower than that of a radiologist performed US [14], when the negative LCUS is repeated after 1 week, it has been shown to be safe to withhold anticoagulation treatment if no thrombosis has been identified. The risk of a thromboembolic complication after two negative LCUS examinations parallels that of a single whole-leg ultrasound, being 0.6% during the 3-month follow-up [6, 14, 15]. It has been shown that using LCUS in primary health care can reduce the number of patients referred to hospital significantly, by 73% [16].

Even though the risk assessment and D-dimer measurement is widely used, approximately 2% of all ED patients undergo a venous ultrasound (Central Finland Central Hospital and Tampere University Hospital statistics). Since the number of patients is large, this is extremely expensive. In a recent review on cost-effectiveness of ultrasound in emergency care setting, point-of-care ultrasound (POCUS) was found to allow for more cost-effective care, although the existing evidence is limited [17]. Otherwise, the data regarding the costs of diagnosing a DVT is scarce. A cost-effectiveness analysis on multiple different diagnostic strategies in a hospital has been previously performed [18, 19]. Verma et al. conducted a cost-minimization analysis and found that incorporating a D-dimer assessment into the diagnostic protocol in hospital setting instead of referring every DVT patient to US saved 24% of costs [20]. We are not aware of any studies which have estimated the overall costs of the LCUS protocol.

For the purposes of the present study, two different diagnostic strategies were created. The first strategy (later “standard strategy”) involves a clinical risk assessment to a D-dimer measurement and if DVT cannot be ruled-out, the patient is referred to the hospital to undergo a radiologist performed US. In the second strategy (later “LCUS strategy”), if DVT cannot be ruled out after the clinical risk assessment and D-dimer assay, an LCUS is performed. If a DVT is found, a treatment is initiated. If the clinical risk is low (Wells 0 or less), a negative LCUS rules out DVT. If the clinical risk is moderate to high (Wells 1 or more), and D-dimer positive, a repeated LCUS is performed after 1 week. If the repeated LCUS is still negative, DVT is ruled out. Both strategies are consistent with national guidelines [21]. The goal of this study was to compare the total costs of these different diagnostic strategies of a suspected DVT using a cost-minimization modeling.

## Materials and methods

### Study design

The cost-minimization modeling was conducted retrospectively. In a previous study, the effect of training LCUS to general practitioners (GP) in Saarikka Primary Care Public Utility (a catchment area of 18,000 inhabitants) was examined. Saarikka has two main health care centres located in northern Central Finland 70 and 105 km from the nearest hospital. In the study, there was a reduction of 73% in hospital referrals (*n* = 60 in year 2014 and *n* = 16 in year 2017) after the adoption of LCUS [16].

**Fig. 1:**
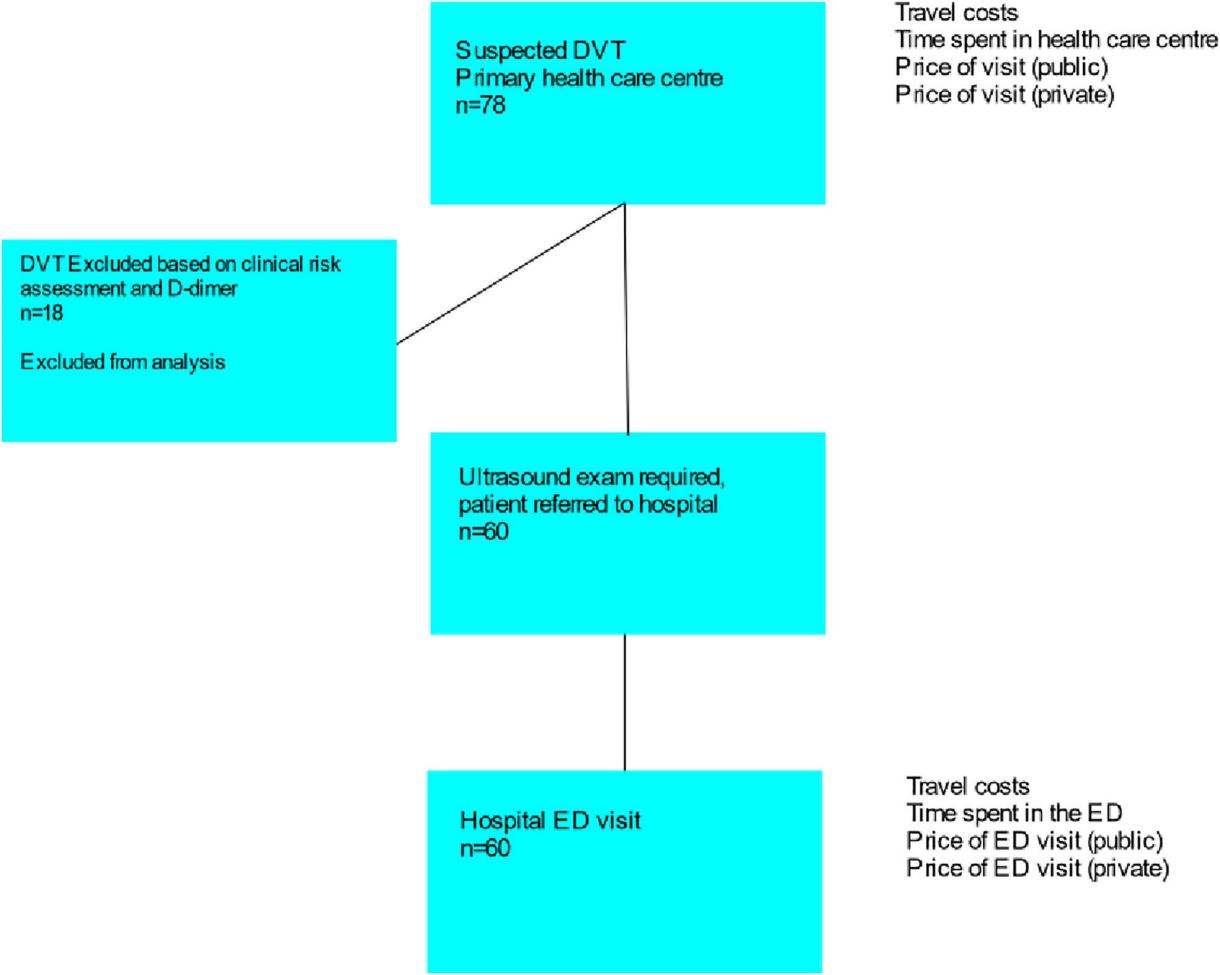
Standard pathway of DVT diagnostics. The costs associated with different parts of the pathway are presented

**Fig. 2:**
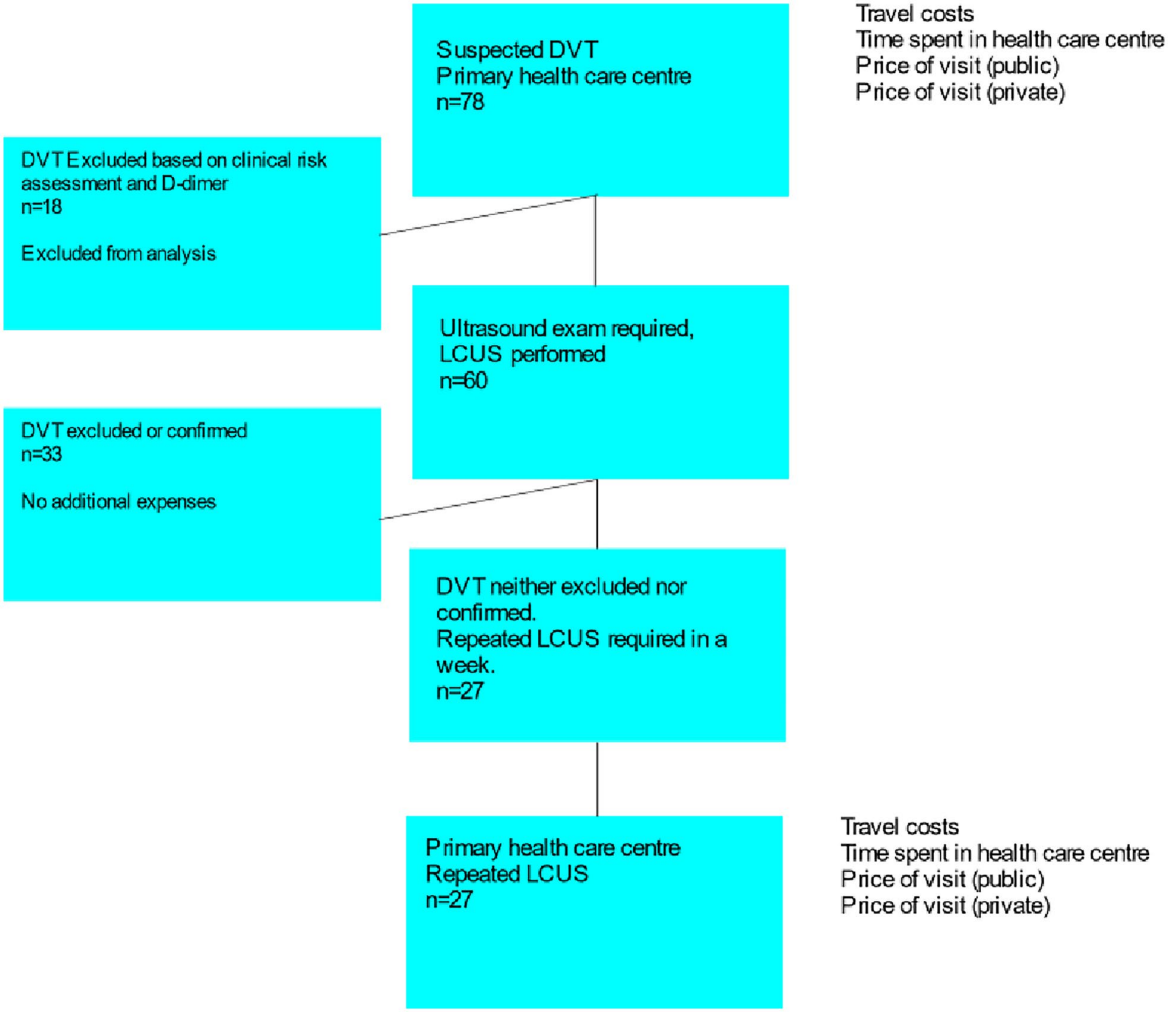
LCUS pathway of DVT diagnostics. The costs associated with different parts of the pathway are presented

In the current analysis, a standardized patient was created based on the measured and estimated average costs incurred by the 76 patients from the previous study. The standard strategy is presented in Fig. 1 and the LCUS strategy in Fig. 2. The costs associated with each strategy are presented in the respective figures. Assuming that 23% of patients had a DVT ruled out with the use of a clinical risk assessment and D-dimer assay [8] and using the number of actual patients from year 2014 (*n* = 60) [16], the estimated number of patients with a suspected DVT was 60/0.77 = 78. As every patient goes through clinical risk assessment and D-dimer measurement to verify the need for US examination, it is safe to assume that every patient referred to hospital had undergone a US examination.

#### Cost analysis

To create the standardized patient, data was collected from the 76 actual patients. This data included home address and time spent in emergency department. The distances from home to the nearest health care centre (average 16 km, 1–49 km), from the nearest health care centre to the central hospital and from home address to the central hospital (average 85 km, 30–131 km) were then calculated. The travelling time was calculated by dividing the distance by 80 km/h, respectively. The time spent in the health care center was approximated as 30 min if referred to hospital (including measuring of D-dimer, 15 min) and 35 min if LCUS was performed and the patient was not referred to the hospital (including 15 min for measuring D-dimer). The average follow-up visit was estimated as 20 min (D-dimer assessment not needed). The length of stay in hospital ED was obtained from the actual patient records and was 3 h 32 min on average.

Measured and estimated times were monetized using the human capital approach and multiplying the time by the average hourly pay 18.70€/h in year 2017 [22]. The travel expenses were calculated for the year 2017 standard taxi fares during the cheapest travel hours [23]. The price of the emergency department visit, 503.45€ in year 2017, was retrieved from the hospital price list (Additional file 1: Appendix S1). This price is used to charge the final payer (local municipalities) for the visit. It represents all costs including imaging, laboratory testing, equipment, and staff. The average primary health care visit price including imaging, laboratory tests, equipment and staff was 96€ in 2011, which is the latest available data [24]. The price paid by the patient for the emergency department visit was 41.20€ [25]. The pricing of a health care centre visit in Finland varies depending on the type of visit and number of visits made in a single year. Since a suspected DVT requires urgent diagnostics and these patients often arrive outside office hours, we decided to use the primary health care urgent appointment price of 28.30€ [25].

Table 1 shows public and private costs. Table 2 shows direct and indirect costs. In Finland, health care visits by a taxi are publicly subsided. Since there was no available data on the patients’ use of taxi throughout the year, all travelling expenses were considered as using this form of public transport.

**Table 1:** Public and private costs

Costs to the public purse	Costs to the individual plus lost working time
Health care centre cost	Health care centre patient fee
Emergency department cost	Emergency department patient fee
Travel expenses	Working time and leisure time lost

**Table 2:** Direct and indirect costs

Direct costs	Indirect costs
Health care centre cost	Working time and leisure time lost
Health care centre patient fee	
Emergency department cost	
Emergency department patient fee	
Travel expenses	

In this analysis, the proportion of patients needing to undergo a repeated LCUS examination was estimated to be 45.9% based on the literature [26–28]. The number of patients requiring a repeated LCUS is difficult to estimate exactly since the patient records are in an unstructured, non-accessible digital format and there is no standardized recording on these examinations. In addition, the Finnish protocol for LCUS is based on the original Wells’ criteria with levels of low, moderate, and high risk [21], whereas the most recent publications have adopted a two-step modified Well’s risk criteria [13, 27, 29]. All patients were assumed to be either in a low-to-moderate risk category with D-dimer positive or alternatively in a high risk, since low-to-moderate risk patients with negative D-dimer should not be referred to any ultrasound examination. It is assumed those patients that have LCUS negative, moderate-to-high risk and who are D-dimer positive will undergo a repeated LCUS.

Finally, the average of all data was calculated to form the standardized patient. The analysis of the two diagnostic pathways was performed using the average patient.

The one-time cost of the training was estimated. The price of the 1-day course, including 2 h of ultrasound physics, machine operating skills, didactic lecture on venous ultrasound and hands-on training on healthy volunteers, was 3500€ for a maximum of 12 participants. The average salary of a GP in Finland in 2017 was 6568€ [30]. This was divided by the average number of working days in a month, 21, and multiplied with the number of participants, 12. Thus the estimated salary cost for the training was 3753€.

The ultrasound equipment is used also for multiple other indications, and therefore, the costs of purchasing the device were excluded from this analysis.

### Cost-minimization modeling

In the cost-minimization modeling, we calculated the total costs of both diagnostic pathways. Subsequently, an additional analysis was made to represent the real-life costs. In this scenario, we calculated the total costs of the standardized patients treated in primary health care (estimated *n* = 44) and patients needing a repeated LCUS (estimated 45.9% of 44, *n* = 20). Most likely, some patients will still need to be referred to hospital for diagnostics. The proportion was estimated to be 27% (*n* = 16) based on the previous literature [16]. The cost of ultrasound training and the salary of participating GPs (*n* = 12) were considered.

### Statistics

The statistical evaluation of the data was based on the Student *t* test for comparing means.

## Results

The total costs of LCUS strategy were shown to be significantly lower than those of the standard pathway therapy (Table 3). Considering the real-life scenario, in which a part of the patients receive the standard strategy, and training LCUS causes expenses, the difference in expenses remains considerable (Table 4).

**Table 3:** Cost calculator demonstrating the cost-minimization modeling on different diagnostic pathways

Costs (€ per patient)	Standard pathway	LCUS pathway	Difference (95% CI)	*p*
Primary health care visit (time, monetized)	9.35	16.86	− 7.51 (− 8.46 to− 6.55)	< 0.001
Primary health care visit price (paid by municipalities)	96.00	139.64	− 43.64 (− 58.34 to – 28.94)	< 0.001
Primary health care visit price (paid by patient)	28.30	41.16	− 12.86 (− 17.20 to − 8.53)	< 0.001
Travel (time, monetized)	45.43	11.08	34.35 (30.32 to 38.38)	< 0.001
Travel expenses	361.73	93.21	268.53 (234.37 to 302.68)	< 0.001
Hospital visit (time, monetized)	66.07	0	66.07 (55.11 to 77.03)	< 0.001
Hospital visit price (paid by municipalities)	503.45	0	503.45 (503.45 to 503.45)	0.001
Hospital visit price (paid by patient)	41.20	0	41.20 (41.20 to 41.20)	0.000
Total costs	1151.53	301.94	849.59 (800.21 to 898.97)	< 0.001
Total costs assuming 60 patients per year	69,091.80	18,116.40	50,975.40

**Table 4:** Cost calculator demonstrating the cost-minimization modeling in real-life including educational costs

Costs (€ per patient)	Standard pathway	LCUS pathway	Difference (95% CI)	*p*
Primary health care visit (time, monetized)	9.35	14.86	− 5.51 (− 6.61 to − 4.40)	< 0.001
Primary health care visit price (paid by municipalities)	96.00	128.00	− 32.00 (− 43.79 to − 20.21)	< 0.001
Primary health care visit price (paid by patient)	28.3	37.73	− 9.43 (− 12.91 to − 5.96)	< 0.001
Travel (time, monetized)	45.43	17.92	27.51 (22.30 to 32.72)	< 0.001
Travel expenses	361.73	148.83	212.90 (169.53 to 256.27)	< 0.001
Hospital visit (time, monetized)	66.07	16.11	49.96 (36.80 to 63.12)	< 0.001
Hospital visit price (paid by municipalities)	503.45	134.25	369.20 (311.20 to 427.19)	< 0.001
Hospital visit price (paid by patient)	41.20	10.99	30.21 (25.47 to 34.96)	< 0.001
Total costs	1151.53	508.69	642.84 (541.85 to 743.82)	< 0.001
Total costs assuming 60 patients per year	69,091.80	30,521.40	38,570.40	
Price of education	0	3500		
Salary of GPs participating in training	0	3753		
Total education cost	0	7253		
Total costs for the first year assuming 60 patients per year including one-time training costs	69,091.80	37,774.40	31,317.40	

Ruling-in or ruling-out lower extremity DVT in primary health care proved to save a significant amount of private and public expenditures. In this study, the costs of the standard diagnostic and LCUS pathways were 1119.45€ and 261.20€, respectively, i.e., a difference of 790.52€ or 75%. Since most likely some patients with suspected DVT are always referred to hospital, another analysis simulating a real-life scenario was performed. In this approach, the cost of the LCUS pathway was 500.19€, with a 619.26€ or 55% reduction in costs per patient. The one-time educational cost of 7253€ is less than the savings made during the first year.

## Discussion

The key finding in this study was that diagnosing or ruling out DVT in primary health care saves private and public resources. Even considering the cost of training, the savings are still substantial. This finding shows that teaching LCUS to GPs could help with the burden of increasing health care costs. As we are living in a world of scarce resources and rising health care costs, it is important to evaluate scientifically where resources can be saved without compromising the quality of care.

These saved resources can be used for the better of the patient, for example hiring more staff. The savings in a relatively small health centre are approximately the same as the salary of a nurse [31]. Furthermore, in a busy emergency department, if there were fewer patients, this would reduce the over-crowding and hence one could argue that the remaining patients would receive better care [32]. Another way of looking at this is that emergency department personnel could concentrate their efforts on those patients that would truly benefit from hospital care.

In addition to the public savings, there are also costs incurred by the individual patient. As the visits to a health care centre consume less time than the visit to the hospital, less working time is lost. The fees paid by the patient are also lower. According to our experience, patient satisfaction is higher when the diagnostics and treatment can be achieved closer to home.

The use of POCUS has been shown to allow for more cost-effective care in emergency care setting [17]. One previous cost-effectiveness analysis of different diagnostic strategies of DVT has been performed [19]. However, that analysis only compared the emergency department expenses when the patient was already in that unit. In that analysis, it was shown that the most cost-effective diagnostic strategy incorporated a clinical risk assessment, D-dimer assay, and ultrasound. Depending on the threshold of willingness to pay for an extra quality-adjusted life expectancy, a repeated ultrasound was recommended.

DVT is a common reason for an emergency department visit. These visits incur significant expenses to both the public and private purses. Although there have been some analysis on parts of the diagnostic pathway [19], no analysis has examined the total expenses including costs to the individual patient. Since the LCUS strategy has been assessed to be as safe as the standard protocol, there was a need for the cost-minimization modeling to demonstrate that it also saves resources.

In this study we performed a cost-minimization modeling of the two strategies that are widely in use. The analysis focused fully on the costs. Since there was no data available on the actual patients diagnosed with the LCUS strategy, we needed to base our analysis on the available data and estimates. The measured data was based on 76 actual patients that had had a suspected DVT. Some data, such as the need for a repeated LCUS, was estimated based on the literature. Since the Finnish guidelines for DVT still use the original Wells’ criteria [21], although most of the recent studies have applied the modified Wells’ criteria, this might have introduced a minor inaccuracy. In an attempt to make the analysis as accurate as possible, despite the inevitable compromises in the estimates that had to be made, comprehensive data was collected including public and private costs.

There are limitations in this study. Since the sensitivity and specificity of LCUS are less than those of US [14], some DVTs are missed and it is possible that these could result in an increase in PEs and obviously any additional PEs would cause significant expenses. However, it has been shown that the number of PEs following the LCUS protocol parallels that with the standard protocol [6, 14, 15]. A possible increase in false positive DVTs would lead to an increase in medical expenses. This increase is assumed to be minor. Since there was no validation by US for the DVTs found to be positive on LCUS, the number of false positives cannot be retrieved from our data and this potential expense has been neglected.

When a DVT is diagnosed, it is often necessary to perform etiologic examinations such as a chest X-ray. The costs of possible additional imaging or laboratory examinations performed during the same visit were not included in the analyses.

This cost-minimization modeling assessed the total costs of traditional and LCUS protocols. The data available for this study was not sufficient for a more patient centered analysis such as cost per life saved or cost per correct diagnosis. An averaged patient was used in the analysis i.e. it was not based on actual individuals, which may introduce some minor inaccuracy. Furthermore, the travel time was estimated using the speed of 80 km/h, which reflects the speed limit of the majority of the roads in the area. Although this might cause a slight underestimate of the travelling time it was considered better than overestimating it. The goal of data collection was to gather as much data as possible and we are confident that the results reflect the actual expenses in Finland fairly well. However, the study was local regarding the organization of the Finnish health care system and the results cannot be straightforward extrapolated to other countries.

## Conclusion

This study using a standardized patient and two diagnostic models shows that diagnosing or ruling out a DVT in primary health care can help save resources. The cost of teaching LCUS to GPs is low in comparison to the potential savings. To reveal the actual costs of the LCUS protocol compared to the standard protocol, a larger prospective multi-center study with LCUS controlled with whole-leg US is needed.

## Supplementary Information

**Additional file 1: Appendix S1.**

## Data Availability

The data sets analysed during the current study are available from the corresponding author on reasonable request.

## Abbreviations

GP: General practitioner
DVT: Deep venous thrombosis
LCUS: Limited compression ultrasound
US: Ultrasound
PE: Pulmonary embolism
CT: Computed tomography
MRI: Magnetic resonance imaging
POCUS: Point-of-care ultrasound
