# Supplementary material for: Cost-minimization modeling of venous thromboembolism diagnostics: performing limited compression ultrasound in primary health care reduces costs compared to referring patients to a hospital
Source: Ultrasound J. 2021 May 27;13:26. doi: 10.1186/s13089-021-00227-5 (PMC8160047; doi:10.1186/s13089-021-00227-5)
Supplement: Supplementary file 1 — Additional file 1: Appendix S1. [file 13089_2021_227_MOESM1_ESM.pdf]

Central Finland hospital district

Patient care prices

Emergency department

Translated from Finnish by Ossi Hannula 1.2.2021.

	Price for member municipalities (€)	Full price (€)
Group 1: nurse practitioner 8.00 – 22.00	49.92	52.42
Group 2: Doctor consultations for primary health care wards, other facilities, pre-hospital care or other authorities	68.49	71.91
Group 3: Visit in emergency department including clinical investigation and point-of-care testing 8.00 – 22.00	70.81	74.35
Group 4: Nurse practitioner 22.00 – 8.00	84.74	88.98
Group 5: Visit in emergency department including clinical investigation and point-of-care testing 22.00 – 8.00	100.99	106.04
Group 6: Visit in emergency department including clinical investigation, point-of-care testing, laboratory or x-ray testing or simple procedures 8.00 – 22.00	164.84	173.08
Group 7: Visit in emergency department including clinical investigation, point-of-care testing, laboratory or x-ray testing or simple procedures 22.00 – 8.00	214.76	225.50
Group 8: Visit in emergency department including assessment of up-to two doctors, more comprehensive examinations (ultrasound or computed tomography) or follow-up over 5h in emergency department	503.45	528.62
Group 9: Visit in emergency department with the need for more than two doctors, extensive examinations by imaging or lab unit, use of expansive drugs or more advanced procedures such as cardioversion or non-invasive ventilation	1257.43	1320.30
